# Durable biventricular assist device support for 1212 days as a bridge to heart transplantation

**DOI:** 10.1093/jscr/rjad372

**Published:** 2023-07-03

**Authors:** Omar M Sharaf, Ahmet Bilgili, Mustafa M Ahmed, Mark S Bleiweis, Eric I Jeng

**Affiliations:** Division of Cardiovascular Surgery, Department of Surgery, University of Florida Health, Gainesville, FL 32610, USA; Division of Cardiovascular Surgery, Department of Surgery, University of Florida Health, Gainesville, FL 32610, USA; Division of Cardiovascular Surgery, Department of Surgery, University of Florida Health, Gainesville, FL 32610, USA; Division of Cardiovascular Surgery, Department of Surgery, University of Florida Health, Gainesville, FL 32610, USA; Division of Cardiovascular Surgery, Department of Surgery, University of Florida Health, Gainesville, FL 32610, USA

**Keywords:** BiVAD, heart transplant, heart failure, mechanical circulatory support

## Abstract

Experience with durable biventricular assist devices (BiVADs) as a bridge to heart transplantation (HTx) is limited, particularly in women. A 41-year-old woman with biventricular failure complicated by cardiogenic shock underwent durable concurrent BiVAD implantation and was supported for 1212 days as a bridge to HTx. During BiVAD support, she experienced bacteremia (day 1030 of support), appropriately managed with intravenous antibiotics. She is alive and well, 1479 days from BiVAD implantation and 267 days from orthotopic HTx. Strategies contributing to successful prolonged support include concurrent BiVAD implantation, aggressive cardiac rehabilitation, diet management for weight loss and frequent interval surveillance.

## INTRODUCTION

Patients with biventricular failure requiring biventricular assist device (BiVAD) support have inferior post-implant outcomes compared to left ventricular assist device (LVAD) patients, with 1-year post-implant survival of ˂60% [1]. Since the need for BiVAD support is expected to increase, understanding strategies associated with successful bridging is important to limit mortality. We discuss the case of a 41-year-old woman presenting with biventricular failure complicated by cardiogenic shock, supported with durable concurrent BiVAD for 1212 days as a bridge to heart transplantation (HTx).

## CASE PRESENTATION

A 41-year-old woman with nonischemic cardiomyopathy, paroxysmal ventricular tachycardia status post implantable cardioverter defibrillator, and left and right heart failure (ejection fraction, 15%) was admitted with cardiogenic shock (INTERMACS profile 2). Right heart catheterization (RHC) revealed elevated right atrial pressure (10 mmHg), pulmonary artery pressure (PAP, 45/25 [mean 34] mmHg) and pulmonary capillary wedge pressure (PCWP, 30 mmHg). Pulmonary artery pulsatility index was 2.0. Cardiac index (CI) was low (1.3 L/min/m [2]). She also had renal dysfunction (creatinine: 2.3 mg/dL; baseline: 1.6 mg/dL). She was managed with dual inotropic support and an intra-aortic balloon pump (IABP) while undergoing evaluation for advanced heart failure therapies.

**Figure 1:**
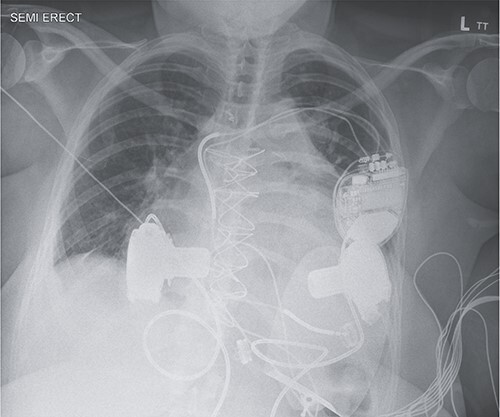
Anterior–posterior chest X-ray following BiVAD implantation.

Given her morbid obesity (body mass index, 40.5 kg/m [2]), she was not a HTx candidate. The Medical Review Board consensus was for concurrent BiVAD implantation as a bridge to possible transplant candidacy. After 19 days with an IABP, she underwent concurrent HeartWare LVAD and HeartWare right ventricular assist device (RVAD) implantation (Fig. 1). LVAD implantation was achieved via the left ventricular apex with the outflow graft sewn to the ascending aorta, and RVAD implantation was achieved via right atrial cannulation with the outflow graft sewn to the main pulmonary artery. Anticoagulation was achieved with warfarin (International Normalized Ratio goal: 2.5–3.5). Her hospital course included persistent epistaxis managed with bilateral sphenopalatine artery ligation, with an otherwise uncomplicated discharge home 30 days post-implantation.

During VAD support, she was readmitted twice—once for 1 day due to acute-on-chronic anemia and the second for 13 days due to methicillin-sensitive *Staphylococcus aureus* bacteremia after 1030 days of support. She did not experience other VAD complications, including pump thrombosis, gastrointestinal bleeding, stroke or renal failure. RHC 1149 days after BiVAD initiation revealed improvements in PAP (29/19 [mean 22] mmHg), PCWP (10 mmHg) and CI (2.5 L/min/m [2]). After 1212 days on BiVAD, she underwent orthotopic HTx with an uncomplicated postoperative course. Serial post-transplantation endomyocardial biopsies have shown no rejection (ISHLT Grade 0). She is alive and well at latest follow-up, 1479 days from BiVAD implantation and 267 days from HTx.

## DISCUSSION

Most patients with end-stage heart failure requiring mechanical circulatory support receive LVAD support only [2]. However, some LVAD candidates at risk for right ventricular failure may benefit from BiVAD support, as right ventricular failure following LVAD implantation is associated with poor outcomes [3]. Identifying strategies for successful prolonged BiVAD bridging is important to improve patient outcomes.

To our knowledge, the longest duration of BiVAD support as a bridge to HTx was 1655 days in a male patient in Croatia [4]. He was supported with sequential HeartMate III implantation and experienced subarachnoid hemorrhage and a new motor deficit along with RVAD infection after several years of support—he was placed on ‘high-urgent’ transplant status and underwent HTx. The authors conclude that in hindsight, they should have opted for concurrent BiVAD implantation due to the risks associated with sequential implantation [2]. Our group has reported success with concurrent HeartMate III implantation for biventricular failure as a 282-day bridge to heart–kidney transplantation [5]. The patient remained in ventricular fibrillation while supported, demonstrating that BiVAD support obviates the need for normal rhythm.

BiVAD implantation is infrequently performed in women given anatomic limitations—a smaller anteroposterior thoracic diameter (compared to men) limits the ability to implant two intrathoracic VAD devices. In fact, over 88% of BiVADs in the last decade have been implanted in men [2]. However, successful long-term, sequential BiVAD support (HeartWare VAD) as a bridge to transplantation in a woman lasting 262 days, without significant complications has been reported [6]. Our case also highlights the feasibility of BiVAD support in women. Specifically, we suspend the RVAD toward the sternum by suturing the device around the 3rd to 4th rib, which may help mitigate anatomic concerns. Nonetheless, one should assess the anteroposterior diameter on a case-by-case basis to determine whether an individual’s thoracic cavity can accommodate two VADs.

Right atrial cannulation versus right ventricular cannulation (in BiVAD support) is associated with improved outcomes [7]. A systematic review demonstrated higher survival with right atrial HeartWare HVAD compared with right ventricular HVAD [8]. These benefits include additional space in the right pleural cavity, reduced risk of VAD ingestion of the tricuspid valve, and decreased incidence of pump thrombosis [7]. In agreement with current literature, we believe that right atrial cannulation in conjunction with concurrent BiVAD implantation contributed to the high quality and prolonged period of life our patient experienced while awaiting transplantation.

In this report, we highlight that long-term survival on BiVAD is possible with concurrent implantation, right atrial cannulation, aggressive cardiac rehabilitation, diet management for weight loss and frequent interval surveillance. While this patient was supported with HeartWare VAD devices, our current strategy includes biventricular HeartMate III implantation. She was supported for 1212 days and experienced methicillin-susceptible staph bacteremia after 1030 days of support. She experienced no other VAD complications and remains alive through latest follow-up, 1479 days after BiVAD initiation and 267 days after HTx.
